# Patient Discomfort Associated with the Use of Intra-arterial Iodinated Contrast Media: A Meta-Analysis of Comparative Randomized Controlled Trials

**DOI:** 10.1186/1471-2342-11-12

**Published:** 2011-05-24

**Authors:** Peter A McCullough, Patrizio Capasso

**Affiliations:** 1 St. John Providence Health System, Department of Medicine, Cardiology Section, Providence Park Heart Institute, Novi, MI, USA; 2 Department of Radiology, Division of Vascular & Interventional Radiology University of Kentucky, Lexington, KY, USA

**Keywords:** contrast, discomfort, pain, intravascular, meta-analysis

## Abstract

**Background:**

Discomfort characterized by pain and warmth are common adverse effects associated with the use of intra-arterial iodinated contrast media (CM). The objective of this review was to pool patient-reported outcomes available from head-to-head randomized controlled trials (RCTs) and to compare the discomfort rates associated with iso-osmolar contrast media (IOCM; i.e., iodixanol) to those reported with various low-osmolar contrast media (LOCM).

**Methods:**

A review of the literature published between 1990 and 2009 available through Medline, Medline Preprints, Embase, Biological Abstracts, BioBase, Cab Abstracts, International Pharmaceutical Abstracts, Life Sciences Collection, Inside Conferences, Energy Database, Engineering Index and Technology Collection was performed to compare rates of discomfort associated with the use of the IOCM (iodixanol) vs. various LOCM agents in head-to-head RCTs. All trials with a Jadad score ≥2 that reported patient discomfort data following intra-arterial administration of CM were reviewed, coded, and extracted.

**Results:**

A total of 22 RCTs (n = 8087) were included. Overall discomfort (regardless of severity) was significantly different between patients receiving IOCM and various LOCMs (risk difference [RD] -0.049; 95% confidence interval [CI]: -0.076, -0.021; p = 0.001). IOCM was favored over all LOCMs combined with a summary RD value of -0.188 (95% CI: -0.265, -0.112; p < 0.001) for incidence of pain, regardless of severity. A greater reduction in the magnitude of pain was observed with IOCM (iodixanol), particularly with selective limb and carotid/intracerebral procedures. Similarly, the meta-analysis of warmth sensation, regardless of severity, favored IOCM over LOCMs with an RD of -0.043 (95% CI: -0.074, -0.011; p = 0.008). A positive linear relationship was observed between the discomfort effect size and age and a negative relationship with increasing proportion of women. The opposite trends were observed with warmth sensation.

**Conclusions:**

IOCM was associated with less frequent and severe patient discomfort during intra-arterial administration. These data support differences in osmolality as a possible determinant of CM discomfort.

## Background

Iodinated contrast media (CM) are essential to intravascular imaging procedures utilizing ionizing radiation. The development of CM has progressed from high-osmolar contrast media (HOCM) with osmolality (particle concentration in milliosmoles per kilogram of water) of ~2000 mOsm/kg, to low-osmolar contrast media (LOCM) with a range of ~600-800 mOsm/kg, to iso-osmolal contrast media (IOCM) at 290 mOsm/kg that is isotonic to blood [1]. The intensity and frequency of adverse-effects associated with intravascular CM injections were reduced considerably with changes in usage from HOCM to LOCM. Nevertheless, patient discomfort during the intravascular administration remains a clinical challenge [2].

More than a third of patients in controlled clinical trials have been known to report CM-injection-related discomfort, particularly local pain and an intense, unpleasant sensation of warmth [3]. The degree of discomfort and tolerability, generally considered to be directly proportional to the osmolality of CM, can influence the quality of the examination. Pain and discomfort may cause patients to move, thus resulting in motion artifacts and suboptimal images. Thus, it is of clinical value to further improve patient comfort and the diagnostic quality of radiological images [4].

Practice recommendations and guidelines issued by national societies have focussed on the risk of renal and cardiac complications after contrast and have not considered potential differences in pain and discomfort [5]. Likewise, most reviews and meta-analyses available in the literature have reported on contrast-induced acute kidney injury as the outcome of interest [1,6-9]. Patient-reported subjective outcomes are infrequently reported in the radiology literature [10]. It is widely believed by radiologists that iso-osmolal contrast causes less discomfort that higher osmolar contrast media; there has not been definitive evidence to support this notion. Therefore, the goals of the current study were to pool data available from head-to-head randomized controlled trials (RCTs) and compare the frequency and severity of discomfort associated with IOCM (iodixanol) to those reported with various LOCM agents.

## Methods

### Search Strategy

A comprehensive search of the literature published from 1990 to Aug 2009 was performed by Nerac Inc. (Tolland, CT) using the following search string: (Visipaque*; iodixanol; 92339-11-2\RN\AL\SU\TM) and (tolerab*; tolerat*; *comfort; warmth; pain*; heat; warm\AL) and (double blind*; double-blind*; prospective; randomized; randomized; head <2> head; parallel\AL) on August 27, 2009. The search was not limited to English language.

### Study Selection and Data Extraction

The resources utilized along with total number of references located from each resource were: Medline (51); Medline Preprints (4); Embase (56); Biological Abstracts (8); BioBase (2); Cab Abstracts (1); International Pharmaceutical Abstracts (0); Life Sciences Collection (4); Inside Conferences (0); Energy Database (5); Engineering Index (1); and Technology Collection (0). Duplicate citations (57) were removed from the search results, and the remaining 75 abstracts were further reviewed for relevance.

**Figure 1:**
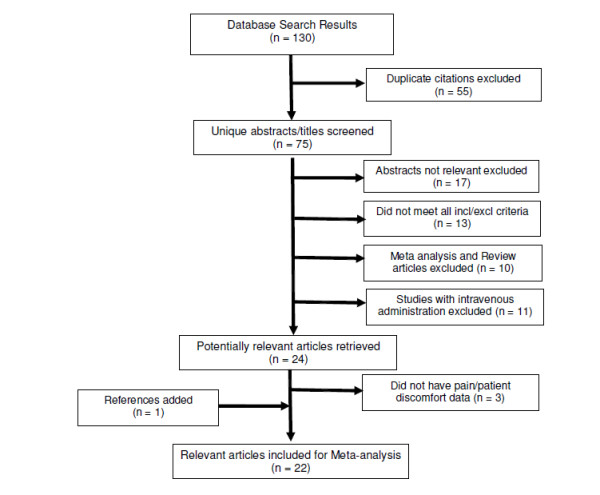
**Flow chart outlining literature search and study selection strategy**.

A flow diagram illustrating the process of elimination used to select set of studies that met the inclusion/exclusion criteria for the meta-analysis is presented in Figure 1. Studies that were not relevant to the topic of research, did not meet all the inclusion criteria, or were either review articles or meta-analyses were excluded. Additionally, studies using intravenous (IV) administration of CM were eliminated since the focus of the current study was limited to studies with intra-arterial (IA) administration of CM.

Data relating to more than 25 parameters were extracted from the RCTs, when available, into a standardized Microsoft^® ^Excel (Redmond, WA) template and were assessed for quality and consistency by independent parties. The validated 5-point Jadad scale was used as an instrument for measuring the quality of each RCT with a score of 5 being indicative of excellent quality on a scale of 0 to 5 [11]. Only studies with a Jadad score of ≥ 2 were included in the meta-analysis.

### Statistical Analyses

Descriptive statistics were reported as means ± standard deviations and counts with proportions as appropriate. Demographic summaries included gender, race, age, and weight for each study population, when available. Baseline characteristics were compared using the Student's t-test, Chi-square, or Wilcoxon rank sum test of the weighted averages, as appropriate.

The risk difference (RD) of pain, discomfort, warmth, and cold between IOCM (iodixanol) and the combined LOCM agents was determined. The first RD analysis was based on the incidence of an event, regardless of severity, while the second RD analysis evaluating severity was performed by grouping no events and mild events together and comparing this group to those with moderate and severe events. A subset analysis was performed between IOCM (iodixanol) and each LOCM agent. A meta-analysis was carried out by using the random-effects model of DerSimonian and Laird to calculate pooled RDs and associated 95% confidence intervals (CIs) for outcomes [12]. This method was chosen because it works independently of heterogeneity and coincides with the inverse variance fixed-effects model if there is no heterogeneity [12].

Statistical heterogeneity of trial results was tested using the Cochran *Q *statistic and *I^2^*, which indicate the percentage of the variability in effect estimates because of heterogeneity rather than chance. For the *Q *statistic, p < 0.10 was considered significant. Subgroup analyses to assess the effect of increasing age and percentage of female patients were performed using meta-regression analysis. Data were also examined for potential publication bias using the Egger and Begg tests as well as funnel plots where RDs were plotted against their corresponding standard errors [13,14]. All statistical tests were 2-sided tests with p < 0.05 regarded as statistically significant. Analyses were done using SAS^® ^(Cary, NC) Version 9.1 and Comprehensive Meta-Analysis Version 2.

## Results

Following the elimination of duplicates from the search results, a total of 75 abstracts were reviewed with 21 articles meeting the predetermined inclusion criteria and one study being added from prior knowledge of the literature as shown in Figure 1. Fifty-one abstracts excluded for the following reasons: 17 studies were preclinical, involved skin testing, hemodynamic assessment, oral hydration, comparative technologies, or had no patient reported outcomes; 13 studies did not use IOCM (iodixanol), were not head-to-head IOCM (iodixanol) versus LOCM, or were not blinded; 11 studies used IV administration; and 10 were meta-analysis and review articles. No attempts were made to contact study authors to either confirm the published trial results or include any unpublished trial results.

The study quality was generally excellent for the RCTs included, with a Jadad score ≥ 2. Most of the articles included a discussion of pain, discomfort or warmth. Some studies were included more than once in the meta-analysis according to the parameter reported, thus the numbers of articles discussed when totaled summed to more than 22 in some instances.

### Characteristics of Reviewed Studies

Table 1 shows an overview of the studies included [15-36]. The patient population sizes for all the reviewed trials, with the exception of three large studies, ranged from 19 to 165 per treatment group; the number enrolled in each of the large studies was greater than 1000 [15-17]. The average age among the 22 trials (n = 8087) was similar between the two groups, 62.5 years for IOCM (iodixanol) and 61.7 years for all LOCMs combined. The proportions of female patients in the IOCM (iodixanol) and LOCM groups were similar (29% and 30.5%, respectively). Although the average body weight, when compared for the individual IOCM (iodixanol) versus LOCM trials appeared to be similar, patients in the pooled IOCM (iodixanol) arms had significantly lower body weight than those in all LOCM groups combined (74.5 kg vs. 80.2 kg, respectively). The individual LOCMs compared in the trials included iohexol, ioxaglate, iopamidol, and iomeprol.

**Table 1:** Study characteristics of RCTs included in the meta-analysis

Study	Injection Site and/or Procedure	Treatment group	Number of patients in comfort analysis	Number (%) of Females	Age (Mean [SD])	Weight (Mean [SD])
Andersen et al[28]	Left ventricle & selective coronary artery injections (left and right)	Iodixanol	36	9 (25.0)	54	81
		
		Ioxaglate	38	13 (34.2)	56	77

Fischbach et al[29]	Celiac trunk/spiral CT angiography of abdominal aorta	Iodixanol	40	10 (25.0)	65.7 ± 11.4	77.1 ± 14.7
		
		Ioversol	38	6 (15.8)	61 ± 11.1	78 ± 11.5

Flinck et al[30]	Cardioangiography	Iodixanol	44	8 (18.2)	62.5	80
		
		Ioxaglate	44	8 (18.2)	62.5	80

Hekster et al[31]	Multiple carotid and vertebral arteries/IA cerebral DSA	Iodixanol	40	16 (40.0)	61	74
		
		Iohexol	39	14 (35.9)	57	71

Hill et al[32]	Coronary and left ventricular angiography	Iodixanol	101	20 (19.8)	61 ± 10.0	unknown
		
		Iohexol	99	12 (12.1)	59 ± 11.0	unknown

Justesen et al[15]	Femoral arteriography	Iodixanol	1225	343 (28.0)	65.6 ± 11.5	71.8 ± 12.5
		
		Iopromide	1227	339 (27.6)	65 ± 11.3	71.7 ± 12.9

Kendall et al[24]	IA cerebral digital subtraction angiography	Iodixanol	44	22 (50.0)	49.7 ± 11.0	70.2 ± 13.5
		
		Iohexol	42	24 (57.1)	45.7 ± 13.3	70.9 ± 13.4

Klow et al[33]	Left ventriculography, selective coronary arteriography, and thoracic aortography	Iodixanol	35	unknown	54 ± 9.0	80 ± 10.0
		
		Iohexol	37	unknown	55 ± 9.0	81 ± 13.0

Manke et al[22]	Femoral arteriography	Iodixanol	163	22 (13.5)	63.6 ± 11.2	74.4 ± 12.9
		
		Iomeprol	165	36 (21.8)	65.2 ± 11.6	74.8 ± 12.6

Manninen et al[34]	IA	Iodixanol	50	24 (48.0)	69	unknown
		
		Iohexol	50	24 (48.0)	69	unknown

Palmers et al[25]	Cerebral arteriography	Iodixanol	40	14 (35.0)	54.9	69.7
		
		Ioxaglate	40	18 (45.0)	48.9	73.9

Poirier et al [18]	Cerebral angiography	Iodixanol	51	unknown	unknown	unknown
		
		Iohexol	48	unknown	unknown	unknown

Pugh et al [19]	Femoral arteriography	Iodixanol	48	12 (25.0)	65 ± 8.9	74.3 ± 14.7
		
		Iopromide	47	13 (27.7)	68 ± 10.4	73.9 ± 12.1

Roriz et al [26]	Left ventricular cardioangiography	Iodixanol	54	13 (24.1)	58 ± 11.0	75 ± 9.0
		
		Ioxaglate	53	13 (24.5)	57 ± 10.0	75 ± 12.0

Rosenblum et al [27]	Peripheral and aortic angiography	Iodixanol	19	13 (68.4)	64 ± 11.0	76 ± 18.0
		
		Ioxaglate	25	9 (36.0)	68 ± 9.0	76 ± 16.0

Siegel et al [21]	Aortography, renal/visceral angiography	Iodixanol	29	11 (37.9)	51 ± 19.0	76 ± 17.0
		
		Ioxaglate	25	9 (36.0)	52 ± 15.0	74 ± 18.0

Singh et al [35]	Abdominal aorta/Abdominal angiography	Iodixanol	39	10 (25.6)	59.1	70.3
		
		Iohexol	20	8 (40.0)	66.1	70.3

Sutton et al [16]	Cardiac catheterization/femoral arteriography	Iodixanol	468	173 (37.0)	60.2 ± 10.0	76.5 ± 14.7
		
		Ioxaglate, Iopamidol	1128	367 (32.5)	60.3	95.1

Sutton et al [17]	Cardiac catheterization/coronary angiography	Iodixanol	665	222 (33.4)	60.7 ± 10.2	78 ± 14.0
		
		Iopamidol, Iomeprol	1443	505 (35.0)	60.4	78.5

Thorstensen et al [23]	Femoral arteriography	Iodixanol	73	31 (42.5)	67.6 ± 12.5	68 ± 12.9
		
		Iohexol	74	33 (44.6)	67.8 ± 11.3	70.2 ± 14.5

Tveit et al [36]	Left ventricular cardioangiography	Iodixanol	53	9 (17.0)	58	76.9
		
		Ioxaglate	49	12 (24.5)	58	77.9

Verow et al [20]	Aorta/Femoral arteriography	Iodixanol	68	unknown	unknown	unknown
		
		Iopamidol	65	unknown	unknown	unknown

**Combined papers**		**Iodixanol**	**3385**	**982 (29)**	**62.5**	74.5*
		
		**All LOCMs Combined**	**4796**	**1463 (30.5)**	**61.7**	**80.2**

*Indicates significant difference between groups (p < 0.05)

### Contrast Media Associated Patient Discomfort

**Figure 2:**
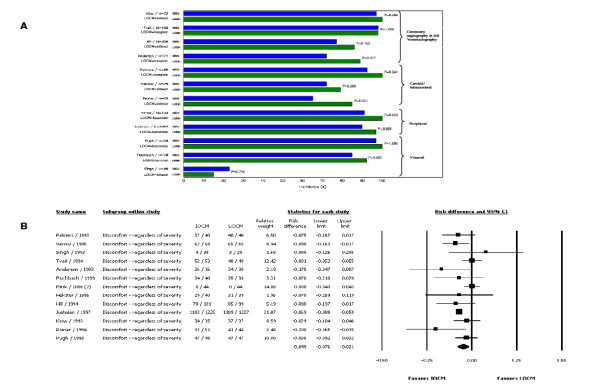
**A. Incidence of any discomfort (regardless of severity) associated with CM injections organized by region**. B. Meta-analysis of incidence of CM-associated discomfort (regardless of severity) - IOCM (iodixanol) vs. LOCMs.

Thirteen of the 22 trials included data on patient discomfort associated with intra-arterial injections (n = 3567) and most of these data did not report severity of discomfort (Figure 2A). In the 2 studies that reported severity, more than 50% of the patient population classified the sensation as mild [18,19]. There was a significant, though small, difference observed in patient discomfort regardless of severity between IOCM (iodixanol) and all LOCMs (the overall RD was -0.049 (95% confidence interval [CI]: -0.076, -0.021; p = 0.001) in favor of IOCM (iodixanol). Heterogeneity across studies was observed (Cochran's Q = 21.247; p = 0.047; I^2 ^= 43.5) (Figure 2B). IOCM (iodixanol) was the favored CM both when the trial data were analyzed individually against each LOCM (ioxaglate, iohexol, iopromide) for discomfort regardless of severity and for all LOCMs combined as a group. The number of studies identified for iopamidol (n = 1) and iomeprol (n = 0) was not adequate to allow the meta-analysis for incidence of discomfort to be performed for these LOCMs. However, the IOCM iodixanol was favored when compared to iopamidol (p = 0.028) [20].

### Patient Reported Pain

**Figure 3:**
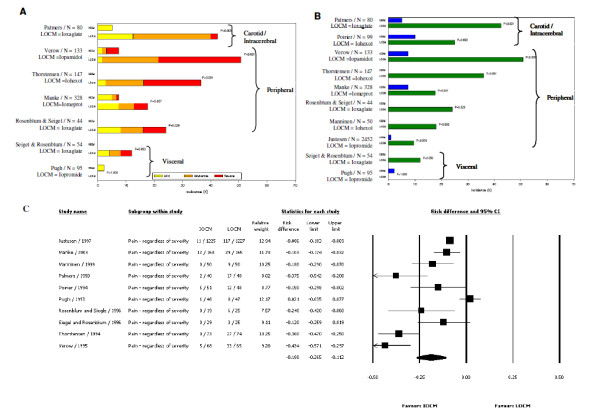
**A. Incidence of CM-associated pain based on severity**. B. Incidence of any pain (regardless of severity) associated with CM injection. C. Meta-analysis of incidence of CM-associated pain (regardless of severity) - IOCM (iodixanol) vs. LOCMs.

Figure 3 presents the incidence of CM-associated pain based on severity in three panels: Panel A illustrates the incidence data on pain, based on severity, reported by a total of 7 trials (n = 881); Panel B illustrates the assessment of any pain associated with CM injections in 10 trials (n = 3482); and Panel C illustrates results from the meta-analysis. Contrary to the observations in patient discomfort studies discussed above, a more pronounced difference was evident in the incidence of pain between IOCM (iodixanol) and LOCM in seven RCTs. The difference in the magnitude of patient-reported pain between IOCM (iodixanol) versus LOCMs (ioxaglate, iohexol, and iopamidol) was greater for peripheral and carotid/intracerebral procedures compared to visceral procedures. Although 3 of the 10 studies included in the analysis reported no statistically significant difference in the incidence of pain (Poirier et al, p = 0.062; Pugh et al, p = 1.000; Siegel et al, p = 0.093) between iodixanol vs. iopromide, iohexol, and ioxaglate, respectively (Figure 3B), our meta-analysis favored IOCM (iodixanol) compared to all LOCMs combined, with a summary RD value of -0.188 (95% CI: -0.265, -0.112; p < 0.001) for incidence of pain, regardless of the severity [18,19,21]. The overall effect size was -0.191, 95% CI: -0.305, -0.077; p < 0.001 when patient-reported incidence of pain was based on moderate or severe vs. none or mild intensity. IOCM (iodixanol) was the favored CM both when the trial data were analyzed individually against each LOCM for which at least three studies were available (ioxaglate and iohexol) and for all LOCMs combined as a group. The number of studies identified for iopamidol (n = 1) and iomeprol (n = 1) was not adequate to allow the meta-analysis for incidence of pain to be performed for these LOCMs. However, the IOCM iodixanol was favored when compared to iopamidol (p < 0.001) and to iomeprol (p = 0.007) [20,22].

### Warmth Sensation

**Figure 4:**
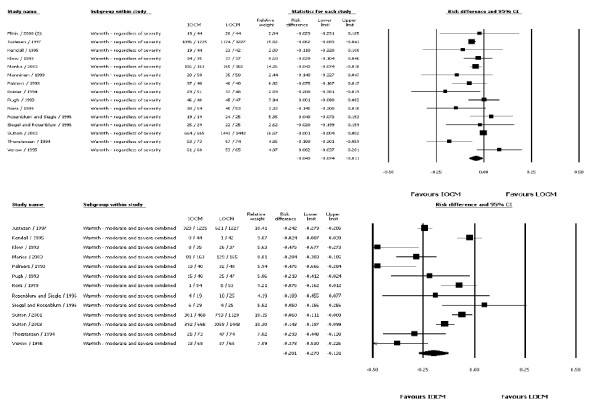
**Meta-analysis of incidence of CM-associated warmth regardless of severity - IOCM (iodixanol) vs. all LOCMs (upper panel)**; Meta-analysis of incidence of CM-associated warmth, moderate or severe vs none or mild severity - All LOCMs (lower panel).

Of the 22 RCTs, 15 (68%; n = 5899) reported the incidence of warmth with injection, and 13 studies (59%; n = 7302) provided information on its severity. Injection-related warmth was reported by more than 90% of patients, irrespective of the CM used. With the exception of three trials, there were no statistically significant differences between IOCM (iodixanol) and LOCMs (ioxaglate, iohexol, iomeprol, iopromide, iopamidol) regarding the incidence of warmth [15,18,23]. One of the two studies that did not report severity data showed a significant difference between iodixanol (57%) and iohexol (77%), p = 0.036 [18]. The meta-analysis of the warmth data modestly favored IOCM (iodixanol) over LOCMs (Figure 4, upper panel) with an effect size of -0.043 (95% CI: -0.074, -0.011; p = 0.008). This effect was more pronounced when the incidence of warmth was evaluated by severity (Figure 4, lower panel). The RD for the latter was -0.201 (95% CI: -0.270, -0.131; p < 0.001). Similarly, when the meta-analysis for incidence of warmth (with or without the severity data) was performed against each LOCM, iodixanol was favored over ioxaglate, iopromide, and iohexol. The three studies comparing iodixanol vs. iopamidol did not favor either agent. No evidence of publication bias was noted among the studies reporting CM-associated warmth.

### Cold Sensation

Only five studies reported data (n = 450) on CM-associated cold sensation experienced by patients undergoing radiological procedures [20,24-27]. Minimal rates (< 5.3%) of mild coldness were reported by patients given IOCM iodixanol. The meta-analysis of CM-associated cold sensation (with or without severity) did not show a difference between IOCM (iodixanol) and LOCM with an overall effect size of 0.008 (95% CI: -0.013, 0.030; p = 0.0449) using a fixed or random effects model.

### Multivariate Meta-regression Analysis

**Figure 5:**
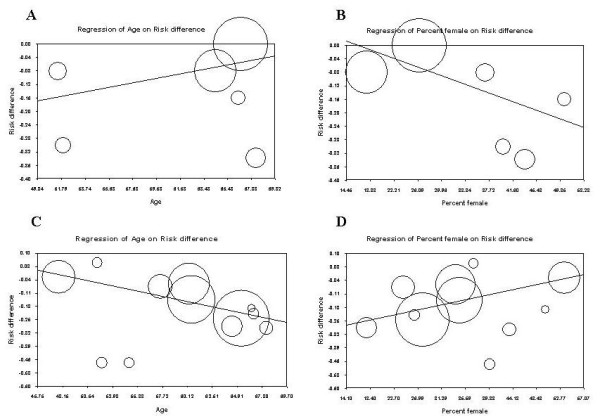
**Bivariate fixed-effects meta-regression analysis with continuous covariates**. The open circles in each panel represent the primary trials. The area of each circle is directly proportional to the relative weight of that study. The solid line is the regression line. (Panels A and B represent the incidence of CM-associated pain with severity; Panels C and D represent CM-associated warmth with severity).

The results of the meta-regression analyses with RD of each event as the dependent variable and increasing age and proportion of women as the independent variables are presented in Figure 5. With regard to incidence of pain (moderate or severe vs. none or mild), there was a slight increase of RDs with increasing age (Tau-squared = 0.01467, slope = 0.00690 and intercept = -0.5137) (Figure 5, Panel A), whereas the RD tended to decline with and increasing percent of women enrolled in the trials (Tau-squared = 0.0111, slope = -0.00663 and intercept = 0.10932) (Figure 5, Panel B). The opposite age and gender trends were found with warmth sensation as the outcome (Figure 5, Panels C and D) with Tau-squared values of 0.00768 and 0.0078, respectively.

## Discussion

We found that the intra-arterial use of IOCM (iodixanol) compared with individual LOCM agents or LOCM as a group was associated with reduced frequency and severity of pain, warmth, and discomfort reported by patients in prospective, head-to-head, RCTs. Older age was associated with greater effect sizes with respect to pain but lesser effects with warmth. The opposite trends were noted as the proportion of women increased in the trials, suggesting both age and gender modify patient-reported outcomes according to the osmolality of CM. There was a moderate degree of heterogeneity among the trials because of differences in trial design, reporting methods of patient symptoms, and external consistency between the trials. There was no evidence of publication bias, and we do not expect additional trials to overturn the results of this analysis.

Our data are consistent with what is known about the vascular biologic effects of iodinated CM. All forms of iodinated CM position iodine on a single benzene ring or a dimer of such rings. The presence or absence of charged side chains, particle concentration in solution (osmolality), viscosity, and iodine concentrations are the main physiochemical characteristics that make each formulation unique. Iodixanol is an iso-osmolar, nonionic, dimer that is isotonic with blood. It is believed that this formulation results in less deformation of cell membranes in blood and the vascular endothelium. As a result, there is an attenuated immediate release of histamine from basophils and nitric oxide from vascular endothelial cells. Thus, there is a blunted initial wave of vasodilation throughout the body as CM travels through the vasculature. In addition, with iso-osmolality, a less pronounced vasoconstriction is anticipated following the initial phase of endothelium-dependent vasodilation. As a result, there is greater vascular stability in arterioles that serve the skeletal muscles and skin in the extremities. This attenuates the activation of nociceptors in nerves supplying both the neurovascular bundles as well as the end-organs. Because the greatest physiochemical difference between IOCM (iodixanol) and LOCM is osmolality as opposed to iodine content or viscosity, we believe, our data support osmolality being the main determinant of symptoms after intravascular injection.

The clinical importance of our findings is highlighted in the ever increasing use of iodinated contrast for intravascular imaging procedures. Our results extend the observations of Justesen and coworkers whose trial included in our meta-analysis [15]. In this trial alone, 1225 patients were randomized to iodixanol and 1227 to iopromide in conventional/digital subtraction angiography of the femoral arterial system. The iodixanol group reported statistically significantly less injection-associated pain (0.9%) than the iopromide group (9.5%) (p < 0.001). In addition, 4.1% in the iodixanol group experienced pain and/or severe heat sensation vs 19. 8% in the iopromide group (p < 0.001). Our analysis suggests these findings can be generalized to other peripheral arterial beds and left ventriculography.

Reduction in pain and discomfort is an important goal for improving the overall tolerability of any procedure. If symptoms related to CM cause tachycardia or body motion, the procedure may be prolonged and the quality of a variety of imaging tests could be affected. This threatens the diagnostic accuracy and subsequent clinical decision making. In addition, poor image quality because of motion artifact may influence the outcomes of an interventional procedure such as a vascular stent placement planned from digital subtraction angiography. Moreover, non-diagnostic studies often lead to repeated examinations, exposing patients to additional injections of contrast and higher doses of radiation. Thus, for all of these reasons, the choice of IOCM over LOCM would be supported in peripheral arteriography procedures where higher degrees of discomfort or body motion would be expected with injection.

Our analysis has all the limitations of any tabular meta-analysis: the response variables measured, stratifications reported, and the individual trial sample sizes. We did not have information on the rates of injection, bolus size, or the use of power injectors, or the use of conscious sedation and analgesic medications which could have influenced the overall size and concentration of CM moving en bloc through the vasculature and its triggering of nociceptors. Importantly, none of the studies had physiologic correlates such as skin temperature, bioimpedance, or plethysmography to investigate the neurovascular origins of discomfort reported. We had insufficient information on the injection site to draw conclusions on outcomes in typically very sensitive vascular territories (distal upper limb and pudendal artery) as well as on injection rates and CM concentration. We included coronary angiography, which for the most part elicits few symptoms, and thus, biased our findings to the null hypothesis. This being considered, the large effect size, internal and external consistency, and absence of publication bias all suggest the differential findings among the CM are valid and likely to be reproduced in everyday clinical practice. Finally, we did not have data on patient motion and image quality, but we suspect in cases where the discomfort was greater, there was more patient motion and the possibility of reduction in image quality.

## Conclusions

In conclusion, IOCM (iodixanol) is associated with less frequent and severe patient discomfort characterized as pain and warmth during intravascular administration compared to the individual LOCM or LOCM as a group. These data support difference in osmolality as the major determinant of such symptoms with CM use.

## Competing interests

This meta-analysis was supported by GE Healthcare, Inc. Medical Diagnostics, Princeton, NJ, USA. GE Healthcare directly contracted with Nerac Inc., Toland, CT, USA, for access and maintenance of the library of papers and abstracts used in the study. GE Healthcare also directly contracted with i3 Statprobe, Inc., Ann Arbor, MI USA for the services listed above. Drs. McCullough and Capasso have served as consultants to GE Healthcare in the past.

## Authors' contributions

PM directed the study design, literature search, reviewed the findings, directed the coding and scoring of papers, tabular synthesis, meta-analysis, and meta-regression. PM drafted the original manuscript and provided edits and clarifications. PC provided input on the concept of the paper, scope of the clinical applications to be studied, review of the tabular and meta-analytic results, and edits and reviews of manuscript versions. Both authors had full access to the raw data set and take responsibility for the integrity of the data and the accuracy of the analysis, results, and discussion. Both authors read and approved the final manuscript.

## Pre-publication history

The pre-publication history for this paper can be accessed here:

http://www.biomedcentral.com/1471-2342/11/12/prepub
